# Axillary Lymphadenectomy: Safe Dissection Through a Correct Technique

**DOI:** 10.7759/cureus.52434

**Published:** 2024-01-17

**Authors:** Iulian M Slavu, Adrian Tulin, Florin Filipoiu, Alexandru Dogaru, Octavian Munteanu, Oprescu Macovei Anca Monica, Raluca Tulin, Bogdan Ursut

**Affiliations:** 1 Anatomy, Carol Davila University of Medicine and Pharmacy, Bucharest, ROU; 2 General Surgery, Agrippa Ionescu Emergency Hospital, Bucharest, ROU; 3 Obstetrics and Gynaecology, Carol Davila University of Medicine and Pharmacy, Bucharest, ROU; 4 Gastroenterology, Agrippa Ionescu Emergency Hospital, Bucharest, ROU; 5 Anatomy and Embryology, Carol Davila University of Medicine and Pharmacy, Bucharest, ROU; 6 Endocrinology, Agrippa Ionescu Emergency Hospital, Bucharest, ROU

**Keywords:** en bloc breast implant removal, breast disease, breast and endocrine surgery, thoracic and breast oncology - areas of interest, breast conservation therapy

## Abstract

The primary treatment of breast cancer in sentinel-positive ganglia includes axillary lymphatic nodal dissection. The LAD (lymphatic axillary dissection) has decreased in overall numbers but due to the increasing incidence of breast cancer, it is practised on a daily basis, even though there is a myriad of complications such as numbness of the upper limb and chest wall, movement restriction of the upper limb, and chronic pain which appear due to trauma to the nerves which pass through the axilla. However, the utility in the overall survival or DFS (disease-free survival) of the patient is unquestionable.

In our study, through the dissection of cadavers, we exposed the vital structures and the anatomical relations of this region. We aimed to offer a map or technique for the surgeon to follow to decrease the overall morbidity of this procedure.

## Introduction

Breast cancer is the most common type of cancer diagnosed in women globally [1]. Tumor invasion of the axillary lymph nodes represents the most important long-term prognostic factor related to DFS (disease-free survival). In Europe, the incidence in 2023 was 94.2/100,000 and the mortality was 23.1/100,000 [1]. In most Western countries, the mortality rate has decreased in recent years due to improved treatment and early detection [2]. Because imaging techniques have limited sensitivity, the regional lymphatic nodes must be explored surgically. In diagnosed cases of breast cancer, sentinel lymph node biopsy has become the gold standard for diagnosis and surgical treatment [3]. Under these conditions, axillary dissection with associated lymphadenectomy, which until 20 years ago was used electively when breast cancer was diagnosed, no matter the stage or location, is currently limited to situations where the extemporaneous examination is positive for the sentinel axillary lymph node. A very good example in this regard is provided by the Novoa team, which found a reduction of up to 50% in the number of axillary lymphadenectomies over the last 20 years [4]. Sentinel lymph node biopsy significantly reduces postoperative morbidity by reducing shoulder dysfunction, and lymphedema, and at the same time, it permits a high standard of diagnostic accuracy and prognosis. Despite the risk of significant postoperative complications such as shoulder dysfunction or lymphedema, the number of axillary lymphadenectomies remains great when one considers the high incidence of breast cancer[1, 5]*. *A full understanding of the surgical anatomy of the axilla, especially the risk elements as well as their relationships to one another is essential to reduce postoperative morbidity. This knowledge will allow dissection and resection in anatomical planes of the axillary lymph node tissue [6]. The purpose of this article is to make a clear exposition, through cadaver dissection, of the anatomical relationships between different essential structures of the axilla.

## Materials and methods

This anatomical study included the dissection of two cadavers. The dissections were carried out in collaboration with the Anatomy Department of the Carol Davila University of Medicine and Pharmacy, Bucharest, Romania. The preservation of the cadavers was done in approximately 20 liters of a solution containing formalin at a concentration of 10% to obtain a tissue saturation of formalin of 4%. After each dissection procedure, the cadaver was wrapped in formalin-soaked material and then kept in specialized, sealed bags. During the dissection process, the following instruments were used: a dissection kit that included a scalpel, anatomical forceps, surgical forceps, scissors, Kocher forceps, and Pean forceps. The photographs of the dissected structures were taken with a personal camera: a Nikon D700. The study has been approved by the ethical committee with the number 237/14.10.2023

Dissection method

The upper limb was placed in 90-degree abduction with a sandbag under the axillary region for maximum exposure. The incision to expose the axilla was oriented on the medial side of the arm with the removal of the skin in a circumferential manner. The dissection continued with an incision on the lateral border of the pectoralis major muscle up to the seventh rib on the chest. The skin was removed from the lateral aspect of the thorax to the lateral edge of the sternum, simultaneously lifting the skin from the clavicular and supraclavicular region. The superficial thoracic fascia was highlighted, which can be observed to continue with the fascia of the upper limb (inferior) but also with the axillary clavipectoral fascia (superior). The axillary fascia is incised and thus the axilla is opened. The suspensory ligament of the axilla is identified and resected. Afterward, the dissection and removal of the lymphatic and fatty connective tissue from inside the axilla were performed. The vascular and nervous elements and axillary lymph node stations are highlighted. The dissection was continued by removing the mammary gland, thus identifying the space between the two pectoral muscles. Medial to the pectoralis minor, the medial pectoral neurovascular bundle was identified. In the space between the two pectoral muscles, the adipose atmosphere containing the Rotter-interpectoral lymph nodes is identified. In the same region, the axillary vein is described and identified in its suprapectoral segment of the axilla together with the apical lymphatic nodes. The dissection progresses from medial to superior on the edge of the pectoralis major where the axillary vein is identified - the most superficial element of the axillary neurovascular bundle. The dissection along the axillary vein was continued until we intersected with the projection area of ​​the latissimus dorsi muscle. The dissection descends inferiorly in the axis of the subscapular and thoracodorsal neurovascular bundle. At this point, the dihedral angle between the thorax and the lateral edge of the latissimus dorsi muscle is observed. Orientation in the adipose tissue on the medial wall of the axillary region was achieved after the lateral thoracic neurovascular bundle was identified. Within it, the thoracic nerve is intrafascial and better protected - a key element that shields it from trauma.

## Results

Long thoracic nerve

**Figure 1:**
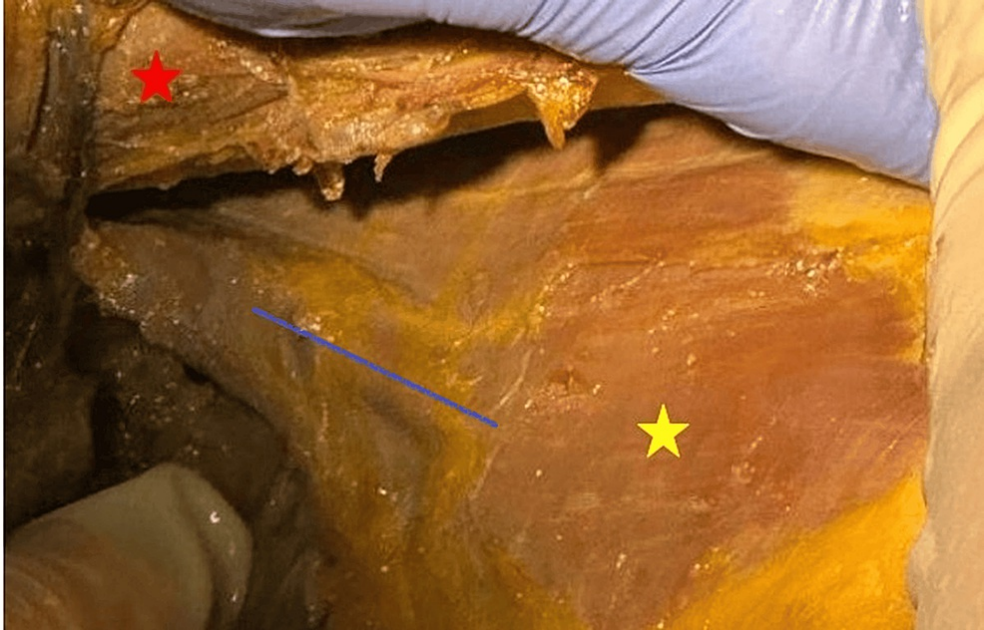
Long thoracic nerve on the medial wall of the axilla after the skin was removed We can observe the trajectory of the long thoracic nerve after the skin and subcutaneous tissue were removed which courses on the medial wall of the axilla, anterior to the serratus muscle (dotted blue line). It is enveloped in a sheath which offers protection against trauma and lesions, thus making this region a good place to start the dissection. Yellow star - lateral thoracic wall covered by the serratus muscle. Red star – large pectoral muscle.

The long thoracic nerve innervates the anterior serratus muscle. It can be observed at the intersection between the axillary vein and the second rib. A meticulous dissection in this area will reveal it has a descending and posterior trajectory to the axillary vein. If we dissect and raise the superficial fascia from the chest wall, the nerve is attached to it and will be raised thus exposing it to trauma. As in the case of the subscapular nerve, it must be identified and protected, except in the cases where there is obvious tumor invasion. The trauma is clinically translated by “winging scapula” in which the scapula protrudes from the back in an abnormal manner, causing weakness in arm movements and impaired shoulder function(Figure 1).

Intercostobrachial nerves

**Figure 2:**
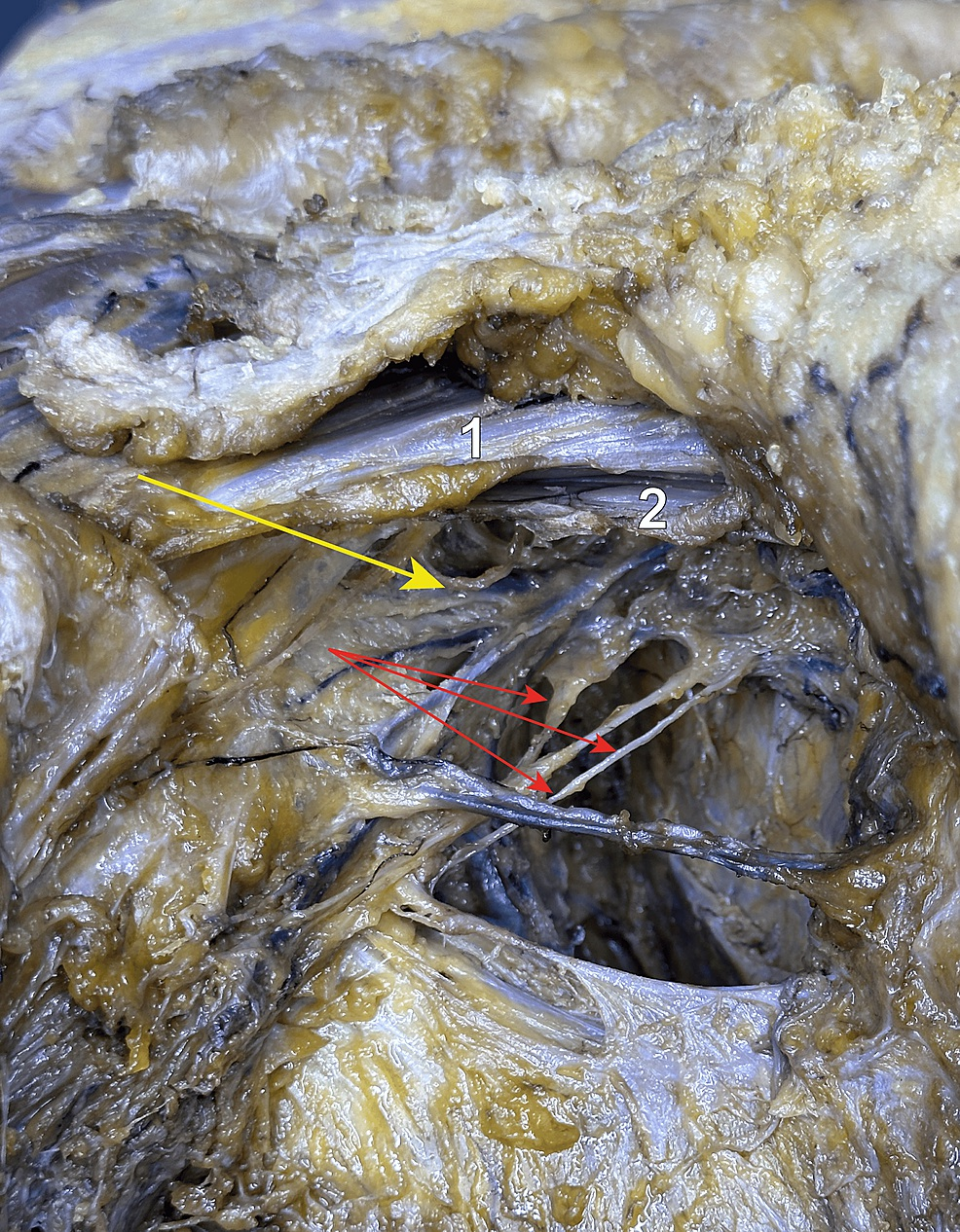
The right axilla is viewed from inferior and lateral We can observe three intercostobrachial nerves, indicated by the red arrows. They provide sensitive innervation to the skin of the axilla, as well as to the skin on the upper medial aspect of the arm. The yellow arrow points towards the basillic vein ending into the axillary vein. 1 - pectoralis major muscle; 2 - pectoralis minor muscle; Red arrows - three intercostobrachial nerves; Yellow arrow - ending point of the basillic vein.

The intercostobrachial nerve is a collateral branch that arises from the second or third intercostal nerve. It may also contain nerve fibers from both intercostal nerves. It is located and follows an oblique path from medial to lateral in the subcutaneous and lymphatic tissue of the axilla. Its branches are distributed to the skin in the medial and upper regions of the arm as well as the armpit. Due to its location and the limited field it innervates, this nerve is frequently sacrificed during axillary dissection (Figure 2).

Thoracodorsal nerve

**Figure 3:**
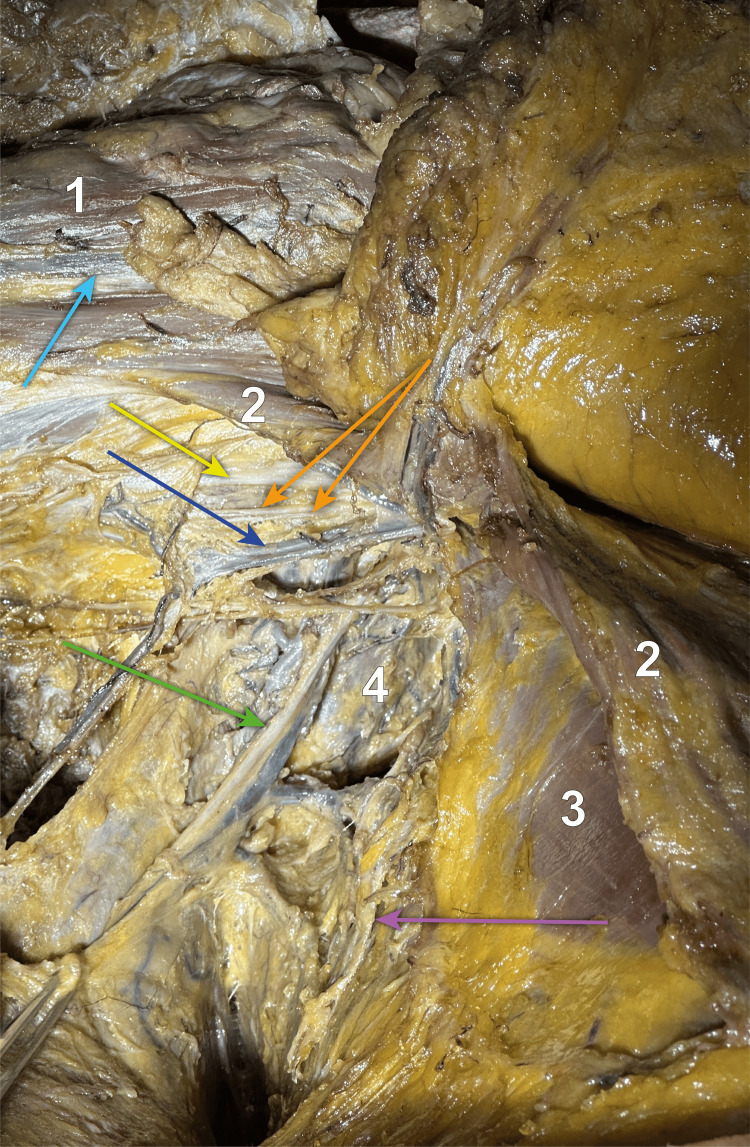
Close-up view of the axilla from inferior to superior We dissected into the axilla, sacrificing the anastomosis between the lateral thoracic and the axillary veins, up to the angle between the serratus anterior and the latissimus dorsi muscles, where we identified and individualised the thoracodorsal neurovascular bundle, showed by the green arrow, that runs between the two muscles. The thoracodorsal vein goes posterior to the nerve and underneath the intercostobrachial nerves and drains into the axillary vein (dark blue arrow). The purple arrow points towards the long thoracic nerve, running anterior to the thoracodorsal neurovascular bundle, along with the lateral thoracic artery and vein. The light blue arrow shows the cephalic vein, running in the deltopectoral groove between the deltoid (1) and pectoralis major (2) muscles. The orange arrows show the medial anterbachial and brachial cutaneous nerves and the yellow one indicates the median nerve. 1 - deltoid muscle; 2 - pectoralis major muscle; 3 - pectoralis minor muscle; 4 - serratus anterior muscle; Light blue arrow - cephalic vein in the deltopectoral groove; Yellow arrow - median nerve; Orange arrows - medial brachial and antebrachial cutaneous nerves; Dark blue arrow - axillary vein; Green arrow - thoracodorsal neurovascular bundle; Purple arrow - long thoracic nerve.

The thoracodorsal nerve arises deep in the axilla from the posterior cord of the brachial plexus, and it is located anterior on the subscapular muscle. It continues a downward path towards the medial aspect of the body. It perforates the latissimus dorsi muscle, which it also innervates. The neurovascular bundle can be identified on the medial edge of the previously mentioned muscle, approximately 3-5 cm above the horizontal plane that passes through the third rib. This neurovascular bundle must be protected, but if there is local tumor invasion, it must be sacrificed in order to obtain an R0 oncological resection. Its damage can cause weakness and up to complete dysfunction of the ipsilateral latissimus dorsi muscle. This event is clinically translated by the limitation of shoulder movements (Figure 3).

Anterior thoracic nerves (pectoral)

**Figure 4:**
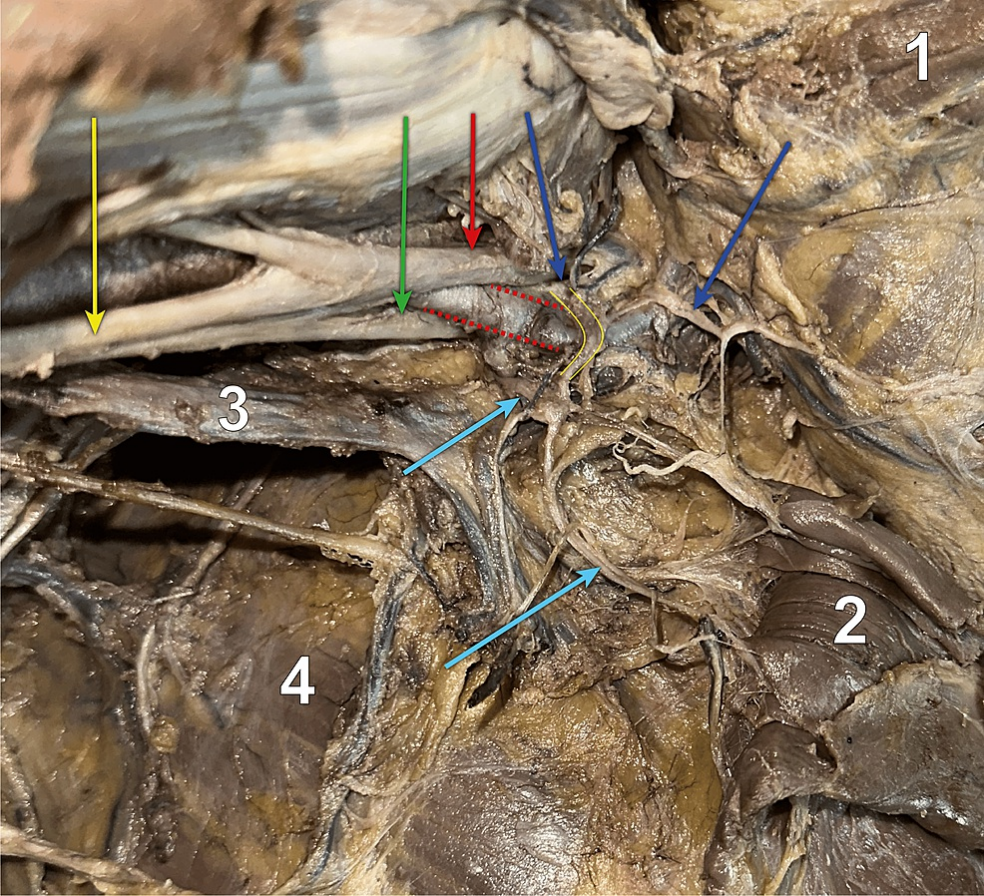
View of the axilla apex after the adipose and lymphatic tissues were removed The axilla after we continued the dissection towards its apex, where we entered by resecting the humeral insertion of the pectoralis minor muscle (2, folded medially). Here we can observe the lateral (red arrow) and medial (green arrow) bundles of the brachial plexus, flanking the axillary artery (red dotted lines follow its pathway), from which a lateral and a medial root emerge and form the median nerve (yellow arrow). Two collateral nerves of the brachial plexus emerge from the lateral and the medial bundles: the lateral (dark blue) and medial (light blue) pectoral nerves. They form an anastomosis that is located anterior and medial to the axillary artery, via the ansa pectoralis, outlined by the yellow lines. 1 - pectoralis major muscle, folded supero-medially; 2 - pectoralis minor muscle, folded medially; 3 - axillary vein; 4 - serratus anterior muscle; Red dotted lines - axillary artery; Red arrow - lateral bundle of the brachial plexus; Green arrow - medial bundle of the brachial plexus; Yellow arrow - median nerve; Dark blue arrows - lateral pectoral nerve; Light blue arrows - medial pectoral nerve; Yellow lines - ansa pectoralis

The pectoral nerves innervate the pectoralis major and pectoralis minor muscles as well as the skin of the anterior region of the chest. We will address two of them that can be encountered in axillary dissection: the medial pectoral nerve and the lateral pectoral nerve. The medial pectoral nerve detaches from the medial cord of the brachial plexus, and is placed near the emergence of the thoracoacromial artery from the axillary artery. In its path, the medial pectoral nerve is initially located superficial to the axillary vein and lateral to the pectoralis minor muscle. The lateral pectoral nerve, which has a larger diameter than the medial one, will innervate both the clavicular portion and the sternal portion of the pectoralis major muscle. The collateral branch of the nerve that innervates the clavicular segment detaches proximal or inferior from the clavicle. The entire nerve trunk follows a superficial route along the axillary vein and is later located on the medial edge of the pectoralis minor muscle. The inferior segment of the lateral pectoral nerve passes anterior to the axillary artery and distal to the origin of the thoracoacromial artery. This crossing point is important because at this level it joins the medial thoracic nerve and forms a nerve loop. From this loop collateral branches emerge that are distributed both to the pectoralis minor muscle and to the pectoralis major muscle which are reached after they perforate the pectoralis minor muscle. These collateral branches will innervate the sternal and costal portion of the pectoralis major muscle. Damage by resection or traction of the pectoral nerves can lead to atrophy of the clavicular head of the muscle but without significantly influencing the motor component. It creates an unpleasant aesthetic aspect. If both medial and lateral pectoral nerves are resected, atrophy of both pectoral muscles will occur (Figure 4).

Axillary vein

**Figure 5:**
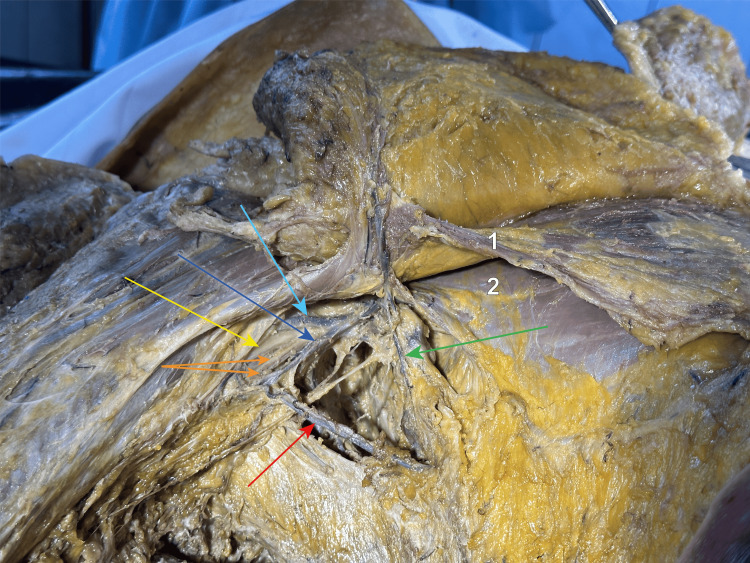
Overview of the axilla, location and surrounding elements We can observe venous anastomoses at the level of the right axilla. Indicated by the dark blue arrow, the axillary vein is the most medial element of the main neurovascular bundle of the axilla and the ending point of the axillary lymphadenectomy that includes the Berg stations I and II. Here we can observe the basilic vein, shown by the light blue arrow, that enters the axilla and ends into the axillary vein. The green arrow points towards the lateral thoracic vein, which lies on the lateral thoracic wall and ends into the axillary vein. The red arrow indicates an anastomotic branch between the lateral thoracic and the axillary veins, running on the base of the axilla. The existence of this branch demonstrates that the venous drainage at the level of the axilla is network-shaped, with numerous collateral drainage pathways that ensure a proper venous flow from the upper limb and from the lateral thoracic wall. The yellow arrow shows the median nerve, while the two orange ones show the medial cut. 1 - pectoralis major muscle; 2 - pectoralis minor muscle; Dark blue arrow - axillary vein; Light blue arrow - basillic vein; Green arrow - lateral thoracic vein; Red arrow - anastomosis between the lateral thoracic and the axillary vein; Yellow arrow - median nerve; Orange arrows - medial brachial and antebrachial cutaneous nerves.

The axillary vein is formed at the lower edge of the anterior serratus muscle. It is located in the apex of the axilla and represents the most superficial element of the axillary neurovascular bundle. The axillary vein drains the entire upper limb and originates from the junction of the basilic veins and the brachial vein. After crossing the lateral edge of the first rib, the name changes to the subclavian vein (Figure 5).

## Discussion

Lymphatic axillary dissection (LAD) has been replaced by sentinel lymph node biopsy as mentioned by the American Society of Breast Surgery [7]. This is required when patients are in the early stages of “cN0”. Keeping this aspect in mind there still exists a large portion of patients who go through this procedure daily and as such anatomical knowledge of the axilla is of utmost importance [7]. The borders are important because they represent a guide that allows a safe orientation to where the dissection evolves and are defined by the following muscles: posterior - the subscapularis, medially by the large serratus, and lateral by the latissimus dorsi. The anterior region is bounded by the clavipectoral fascia and superior by the axillary vein [8]. The inferior boundary of the axilla when the dissection evolves is less well-defined but should reach the axillary tail of the breast [8]. The extent of the dissection needs to take into consideration the distinctive traits of the tumor and intraoperative observations. The dissection must include a minimum of 10 lymphatic nodules for accurate postoperative staging and neoadjuvant therapy although this number has come under scrutiny in recent years [9]. A number of 20 nodes extracted seems to increase the overall survival while above 20 seems to have no impact and should be considered a cut-off point [9].

Three levels of lymphatic nodes are defined in the axilla. The first two levels need to be dissected and extracted. Level 3 nodes are not mandatory for dissection as indicated in the National Comprehensive Cancer Network (NCCN) guidelines for clinical practice [10]. Only if there is gross/macroscopic disease in the level 1 and 2 nodes this third stage should be dissected. Dissection of the third-stage nodes is risky and may lead to paresthesia, lymphedema, or postoperative deformity of the arm and its impact on survival requires further evaluation [11]. The first level lymphatics are located in the lateral region of the small pectoral muscle while the second level nodes reside posterior and deep to the small pectoral muscle. The third-level nodes are placed medial to the small pectoral muscle. To allow safe dissection and avoid the nervous structures in the region, if possible the anesthesia team may avoid paralytic molecules to observe the muscle contraction if a nerve is encountered [11]. 

No LAD leads to a three-fold increase at 5 years of locoregional recurrence which manifests through cancer relapse in the remaining breast tissue in the mastectomy scar or axilla as mentioned by Bromham meta-analysis on 3849 patients [12, 13]. Before the incision, one must identify the boundaries of the axilla which form a triangle under the skin: the upper border is the serratus anterior muscle, the base is the medial aspect of the humerus and the apex is formed by the pectoralis major and latissimus dorsi muscles [14, 15]. The LAD is and should be surgically attached to the mastectomy, completing it or as a single surgical gesture without mastectomy [16]. Taking the above factors into account, the axilla can be approached through the same incision as the mastectomy or through different incisions. When it is included in the same incision as the mastectomy, the main landmark is represented by the lateral edge of the pectoralis major (where the incision on the skin should end).

Penetration into the axilla is carried out towards the upper 1/3 of the incision line by resecting the clavipectoral fascia. The dissection then descends on the medial wall of the axilla until the lateral pectoral neurovascular bundle is identified [17]. This is placed superficial to the fascia of the anterior serratus muscle. Here the lymphatic nodes on the medial wall of the axilla are approached, dissected, and resected [18]. Increased attention at this point of the dissection should be offered to the lateral pectoral neurovascular bundle to avoid iatrogenic lesions. This bundle surrounds and approaches the pectoralis major muscle in the upper and external region of the axilla [19]. The nerve travels with the thoracoacromial artery which can be used as a marker. This bundle travels superficially on the large pectoral muscle and supplies motor innervation to the proximal 1/3 of this muscle [20]. 

The medial pectoral nerve travels through the minor pectoral muscle and innervates 2/3 of the large pectoral muscle. From these aspects, we can observe that when the small pectoral muscle is removed in extreme cases like modified radical mastectomy or there is an inadequate dissection between these two muscles to extract Rotter’s lymphatic ganglia there can be partial denervation of the large pectoral muscle with subsequent atrophy. If also the lateral pectoral bundle is traumatized during the dissection of the mammary gland from the pectoral fascia total denervation of the large pectoral can result in secondary atrophy of the large pectoral muscle [21]. The dissection then continues towards the subscapular muscle. The long thoracic nerve (or Bell) which is located superficial to the anterior serratus muscle, will be observed above the muscle fascia towards the tip of the axilla. This is why the dissection should progress from caudal to cranial, to protect this nerve [22]. 

The next step is to identify the axillary vein that can be identified at the tip of the axilla. This is soft to the touch when compared to the artery and has a somewhat bluish color. It should be dissected inferior and lateral towards the brachial region of the arm. At this point, one can observe the lateral thoracic vein which should be inferior and anterior to the axillary vein and 2-3 cm lateral from the chest wall. It can be used as a marker for the thoracodorsal neurovascular bundle [23]. The dissection descends on the axillary neurovascular bundle without resecting the conjunctive sheath that surrounds the neurovascular bundle. This line of dissection will end where the lateral edge of the latissimus dorsi muscle is felt under the skin. In this way, the lymph nodes on the lateral wall of the axilla are removed without dissecting those in the axillary apex. The neurovascular bundle fascia is kept intact and injuries to the median, ulnar, musculocutaneous, and radial nerves are avoided. The only nerves exposed to injury are the medial brachial cutaneous and medial antebrachial cutaneous nerves that perforate in this area the fascia that surrounds the neurovascular bundle of the arm [24]. Also in this area, we will identify the intercostobrachial nerves that make the connection with the intercostal spaces 2-4. This is the most commonly injured nervous structure in the axilla during LAD. It has been reported that between 10-60% of breast cancer survivors experience upper arm pain and numbness due to trauma of this nerve [25]. They can be kept out of the dissection field or can be resected if the situation requires, such as gross tumor invasion. This can lead to postoperative numbness of the upper arm (medial aspect) [26].

The next step is the dissection of the posterior wall of the axilla. This is achieved by following a downward path from the level of the axillary neurovascular bundle to the base of the axilla. We will identify the subscapular neurovascular bundle from which the circumflex artery of the scapula detaches and will continue with the thoracodorsal neurovascular bundle [27]. The thoracodorsal nerve is usually located posterior to the vascular elements, so it is more protected [28]. This nerve needs to be preserved as it contributes to motor innervation of the latissimus dorsi. Trauma to it may limit the rotation, adduction, or extension of the arm. The nerve can travel inferior and lateral on the subscapular muscle alone for 3 cm and will join the subscapular vessels thus giving rise to the neurovascular bundle. In the bundle, it is easily traced. The landmark for the nerve as mentioned previously is the lateral thoracic vein. The nerve lies underneath it [28]. In this way, we will remove the lymph nodes from the posterior wall of the axilla, which will be resected together with the central axillary nodes.

The dissection will continue in a downward fashion to the angle between the latissimus dorsi muscle and the serratus anterior muscle. In this area, the thoracodorsal neurovascular bundle approaches the latissimus dorsi muscle and needs to be identified and protected as mentioned. If one desires to approach the axilla for dissection through a separate incision from the mastectomy, this will placed transversely at the base of the axilla. Through this incision, the subcutaneous tissue is removed, the concave portion of the clavipectoral fascia will be resected and the surgeon will enter the axilla at the level of the medial wall, following the same guiding steps that we have described in the previous technique. Although largely anatomy is fairly constant in the majority of individuals, some variabilities may be encountered, and this is where the knowledge and experience of the surgeon count. Differences are observed in less vital structures such as the thickness of the subcutaneous adipose tissue which can vary from 8 mm to 60 mm [29]. Other nervous structures such as the medial cutaneous nerve avoid the axilla completely in 22% of individuals [29]. The most constant structure seems to be the neurovascular bundle of the pectoral nerves present in almost 100% of individuals [30]. During LAD, as in all surgical interventions, one must exert caution during dissection.

Limitations

The study used only two cadavers, so a statistical analysis could not be obtained regarding the variable anatomy of the structures. The study reviewed the anatomy only in vitro. It would be of real use and is one of the authors' future desires to review the anatomy of the axilla in vivo, in live surgery, and publish the resulting paper.

## Conclusions

Although 0% morbidity is an impossible desiderate, we as surgeons should strive and search to reduce the rate of postoperative complications. This can be obtained through a complete coherent knowledge of anatomy, what structures to preserve, and what consequences the lesion of these structures can produce.* *The emphasis in modern medicine is on how fast the patient can return to work and back to a normal life. Minimal surgery with maximum results - this paradigm should be followed in breast surgery. If a preplanned plane of dissection is followed, iatrogenic trauma to the noble structure of the axilla can be reduced. Another aspect that should be kept in mind is that the LAD must respect the mastectomy and allow proper skin closure and decent esthetics.
